# Explaining health managers’ information-seeking behaviour and use

**DOI:** 10.1186/1472-6963-14-S2-P34

**Published:** 2014-07-07

**Authors:** Christine Edwards, Pinar Guven-Uslu, Stephen Gourlay, Vari Drennan, Steven Gillard

**Affiliations:** 1 Business School, Kingston University, Kingston, Surrey, UK; 2 Business School, Essex University, Colchester, UK; 3 HSCE Kingston University & St Georges University of London, London, UK; 4 Division of Population Health Sciences and Education, St Georges University of London, London, UK

## 

It is widely assumed that information will reduce uncertainty, help managers make better decisions and bring competitive advantage. Research has largely focused on top level managers in private companies, and on public sector policy-makers, but little is known about managers’ use of information at the organizational level.

The paper addresses this issue by providing empirical evidence to build a theoretical framework to explain managers’ information seeking behaviour. It takes the information science literature as a starting point and elaborates on established theories of generic information search behaviour drawing on research in other disciplines, notably management and organisational behaviour. Data from research funded by the UK National Institute of Health Research included documents and in-depth interviews with 54 managers engaged in major change projects in three hospitals and two service commissioning organisations in the English National Health Service. Managers’ need and search for information may be higher in these settings, especially as many health managers are clinicians trained in “evidence based” practice. Triggers of information need, type of information and sources, as well as the processes of information search and contextual factors are explored to identify an exploratory framework for managers’ information seeking behaviour.

The findings revealed that health managers’ information search behaviour is more complex than generic models would suggest: instead of a sequential process of activities carried out by individuals, the search process was interactive and ongoing with no apparent pattern, beginning or end, and information search and decision making were normally performed by groups. The sources, medium and types of information were many and varied, reflecting the multifaceted nature of managerial tasks. People and informal networks were primary sources, and personal experience and practical demonstrations of “what works” were most used. Research sources were seldom used directly. While contextual factors, notably government policies, strategy and culture (organizational and professional) and task were found to be important, so was agency in the form of managing interests and power relations between stakeholders. Further, there were influential information “leaders” operating at organisational and national level who were sometimes also creators of information as well as users and knowledge brokers. Finally, a research framework identifying the factors salient in the study at individual, group and organizational level is presented.

